# Mechanical Characteristics and Bioactivity of Nanocomposite Hydroxyapatite/Collagen Coated Titanium for Bone Tissue Engineering

**DOI:** 10.3390/bioengineering9120784

**Published:** 2022-12-08

**Authors:** Diana Julaidy Patty, Ari Dwi Nugraheni, Ika Dewi Ana, Yusril Yusuf

**Affiliations:** 1 Department of Physics, Faculty of Mathematics and Natural Science, Universitas Gadjah Mada, Yogyakarta 55281, Indonesia; 2 Department of Physics, Faculty of Mathematics and Natural Science, Universitas Pattimura, Ambon 97233, Indonesia; 3 Department of Dental Biomedical Sciences, Faculty of Dentistry, Universitas Gadjah Mada, Yogyakarta 55281, Indonesia; 4 Research Collaboration Center for Biomedical Scaffolds National Research and Innovation Agency of the Republic of Indonesia (BRIN) and Universitas Gadjah Mada (UGM), Bulaksumur, Yogyakarta 55281, Indonesia

**Keywords:** bioactivity, compressive strength, titanium, hydroxyapatite, collagen

## Abstract

In the present study, we have analyzed the mechanical characteristics and bioactivity of titanium coating with hydroxyapatite/bovine collagen. Hydroxyapatite (HAp) was synthesized from a Pinctada maxima shell and has a stoichiometry (Ca/P) of 1.72 and a crystallinity of 92%, suitable for coating materials according to ISO and Food and Drug Administration (FDA) standards. Titanium (Ti) substrate coatings were fabricated at HAp concentrations of 1% (Ti/HAp-1) and 3% (Ti/HAp-3) and a bovine collagen concentration of 1% (Ti/HAp/Coll) by the electrophoresis deposition (EPD) method. The compressive strength of Ti/HAp-1 and Ti/HAp-3 was 87.28 and 86.19 MPa, respectively, and it increased significantly regarding the control/uncoated Ti (46.71 MPa). Furthermore, the Ti/HAp-coll (69.33 MPa) has lower compressive strength due to collagen substitution (1%). The bioactivity of Ti substrates after the immersion into simulated body fluids (SBF) for 3–10 days showed a high apatite growth (Ca^2+^ and ${\mathrm{PO}}_{4}^{3-}$), according to XRD, FTIR, and SEM-EDS results, significantly on the Ti/HAp-coll.

## 1. Introduction

According to a study by the Royal College of Surgeons in England, bone is the second most frequently transplanted tissue in the body, and more than 3.5 million bone graft procedures are carried out in the UK annually [1]. Bone tissue engineering is aimed at creating a scaffold that mimics natural tissue and ensures the successful regeneration of damaged tissue to restore its normal function. Therefore, the composition and structure of bones are very important for understanding. The bone is a vital organ with an important role in human physiology, consisting of a major mineral phase of calcium(Ca^2+^) phosphate (${\mathrm{PO}}_{4}^{3-}$) with a small part of carbonate (${\mathrm{CO}}_{3}^{2-}$). The organic matrix is about 40% of the volume of bones, consisting of 90% collagen type I and noncollagenous proteins (i.e., osteocalcin, osteonectin, osteopontin) important in bone structure and metabolism; the water component is about 20% of the bone volume [2].

Titanium is a widely used orthopedic implant material due to its low density and excellent corrosion resistance. The development of biocomposites based on HAp and Ti improved their physical properties and biocompatibility, and modern biocomposites are a better alternative to bone replacement. HA-Ti has similar physical properties to the cortical bone in terms of hardness, modulus of elasticity, and fracture toughness. The biomineralization and quantification of the osteoblast cell fate process in these composites were also examined. Routine biochemical analyses used to determine cell viability cannot predict whether the mode of cell death is apoptosis or necrosis; therefore, other molecular biology techniques are being used, such as Fluorescence-activated cell-sorting analysis (FACS), which uses single-cell-based fluorescent detection [3].

HAp is a bioactive and biocompatible mineral of calcium-phosphate, applied as a bone graft, with a chemical composition almost identical to the bone mineral component. The HAp present in the human body is not pure HAp (Ca_10_ (PO_4_)_6_(OH)_2_, and it contains carbonates [4]

HAp is used instead of artificial bones because of its biological properties, including biocompatibility, bioaffinity, bioactivity, osteoconduction, osteointegration, and osteoinduction [5]. When implanted in the body, the newly formed bone binds directly to the HAp through a layer of calcium carbonate deficiency at the bone-implant interface. To determine the bioactivity of the growth of apatite on the surface of HAp using the in vitro method of simulated body fluids (SBF) was developed by Kokubo (1990) (Table 1).

Due to its similarity of chemical composition to bone minerals, hydroxyapatite exhibits excellent biocompatibility and osteoconductive, allowing bone cells to grow on its surface. The key properties of HAp coating for orthopedic implants include low porosity, strong cohesive strength, good adhesion to the substrate, high crystallinity level, chemical purity, and phase stability [6].

Commercial HAp is quite expensive if used in scaffold fabrication. However, HAp bioceramics can be synthesized with a simple synthesis method through solid-state reaction, co-precipitation, hydrothermal, or sol-gel process methods. Biogenic materials containing large amounts of calcium carbonate, such as mammalian bones, shells [7,8,9,10], corals, and eggshells, are used in the synthesis. For example, *Pinctada maxima* shells have been applied for almost a decade in biomaterial research for orthopedic implants [11,12,13]. *P. maxima* has a special layered microstructure and organic components, providing better compressive stress properties than bone. Young’s modulus is 30–40 GPa for nacre versus 20 GPa for bone [14]. Pearl shell (*P. maxima*) is a biogenic material high in calcium carbonate, and this material is abundant and has a significant potential for use in the synthesis of HAp for the reconstruction of bone tissue [15].

Type-I collagen is the main structural protein in bone, having beneficial properties related to bone regeneration. In addition, the collagen scaffold is a well-known cell adhesive protein that effectively promotes cell adhesion and dispersion but has a high degradation rate and will impact the mechanical properties. As a biological composite material for bone function, the interaction of collagen and HAp is essential. The application of collagen in bone graft fabrication must pay attention to temperature treatment because high-temperature treatment can cause damage to the protein in collagen.

Previous research using HAp/Coll on Ti coatings was widely carried out using the spin coating method, immersion in SBF solution [17], and the sintering method of titanium mesh [18]. However, electrophoresis deposition (EPD) is an exciting method for forming biomedical implants. The advantages of the EPD method for the HAp coating process are the good sinterability and uniformity of the precipitate, the possibility of impregnation of porous substrates, and the consolidation of composites [19]

**Figure 1:**
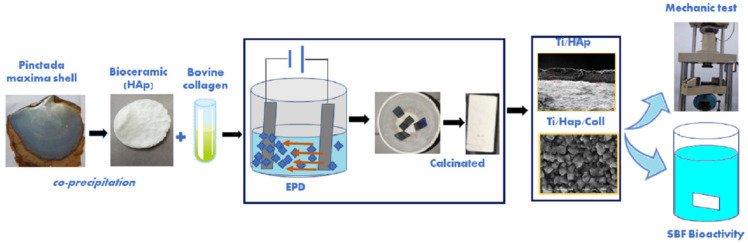
Schematic procedures of coated titanium substrate using HAp/Coll with the mechanical and bioactivity assay.

However, coating Ti/HAp/Coll using the EPD method requires a high-temperature treatment and will denature the collagen. The coating, Ti/Hap, with and without collagen to support the growth apatite, is investigated. According to Figure 1, the experimental result using the EPD method of the coated Ti substrate’s mechanical properties and bioactivity assay are reported.

## 2. Materials and Methods

### 2.1. Materials

HAp bioceramics synthesized from *P. Maxima* shells are biologically active calcium phosphate bioceramics commonly used to coat orthopedic implants or as a bone replacement material. Bioactive HAp promotes bone growth along its surface, and it is an essential material for biomedical implants since its chemical composition is similar to the mineral phase of the bone [20].

The HAp applied on the Ti substrate is synthesized from the *P. maxima* shell using the precipitation method. The collagen type-1 (bovine) was purchased from the Distributor co id Teknoboga, Central 116 Jakarta. Ti substrates (ASTM B265-GR.1) from NET ARTIDAYA, Engineering and Industrial Support Company, Bekasi, Indonesia. SBF solution was prepared according to a published protocol [16] with components from Merck (Table 1) purchased at LPPT Universitas Gadjah Mada (UGM) Yogyakarta.

### 2.2. Synthesis and Characteristics of HAp

The preparation and synthesis of HAp are based on previous studies [21]. Briefly, HAp synthesis by the precipitation method was carried out using CaO extracted from the *P. maxima* shell. First, 2.14 g CaO was dissolved in 50 mL distilled water and stirred at room temperature. Next, 3.56 g phosphate ([NH4]2HPO4 in 50 mL distilled water was dissolved and titrated into CaO solution at 2 mL/min at 90 °C while maintaining pH > 9. Finally, the HAp slurry was aged (24 h) at room temperature and rinsed with distilled water. The HAp product was calcination at 1000 °C for 2 h and characterized using XRF, FTIR, XRD, and SEM.

X-ray fluorescence (XRF) measurements were carried out on a RIGAKU-NEX QC + QuanTEZ XRF, <160 eV Mn K-alpha line detector to analyze the composition and concentration of calcium and phosphate contents in samples to determine the stoichiometry (Ca/P).

Fourier transforms infrared spectroscopy (FTIR) Thermo Nicolet iS10-Japan was used to analyze the functional groups of HAp and Ti substrate before and after SBF immersion. All spectra were recorded between the wavenumber of 4000 and 600 cm^−1^.

Phase analysis of HAp coating and Ti substrates before and after the SBF immersion was conducted using an X-ray diffractometer (XRD)-PAN analysis X’Pert Pro-Japan Type with Cu-Kα radiation (wavelength of 0.154 nm). The data were collected in the 2θ range of 20–80°, and the diffraction peaks were identified using JCPDS data.

The morphology of HAp and the microstructure surface of Ti/HAp and Ti/HAp/Coll before and after SBF immersion was examined by scanning electron microscopy (SEM) Joel JSM-6510LA- 1400-Japan.

### 2.3. Coating Ti substrates by EPD Method

#### 2.3.1. Ti Metal Preparations

Ti substrates (20 × 10 × 0.8 mm^3^) were etched in 12M HCL(10 mL) under heating with a stirrer at 80 °C for 1 h, and then, they were rinsed with distilled water and dried at room temperature.

#### 2.3.2. Preparation of Solution for EPD

The Ti substrate coating used the EPD method, which uses HAp (1% and 3%) and collagen 1%. The solution of coating Ti/HAp substrate using a 0.44 g HAp was dissolved in 40mL distilled water and stirred up to homogeneity. Furthermore, the suspension for Ti/HAp/Coll substrate, using 0.44 g HAp and 0.14 g collagen bovine (in 50 mL distilled water).

Ti substrate coating was prepared using two Ti substrates as cathode and anode. Ti substrates were immersed into Ti/HAp-1 and Ti/HAp-3 HAp solution while stirred gently with a magnetic stirrer (Thermo Fisher Scientific, Waltham, MA, USA) under 50 V for 10 min. Furthermore, other Ti substrate was treated in the HAp/Coll suspension and designated as Ti/Hap/Coll. The Ti/HAp-1, Ti/Hap-3, and Ti/HAp/Coll substrates were dried at room temperature, then calcined in a furnace at 900 °C for 3 h. High-temperature treatment effect denaturing of collagen in Ti/HAp/Coll, and the sample code was changed to Ti/HAp-coll.

### 2.4. Compressive Strength Testing and Statistical Analysis

The mechanical characteristics to analyze the compressive strength of the samples were determined using a universal testing machine (TN20MD, Controlab, Paris, France) at the UGM engineering laboratory. The samples are compressed along their long axis at 2 mm/min compression speed until they break. The force and displacement are recorded throughout compression and converted into stress and strain based on the dimensions of the initial samples. All compressive strength data were presented in mean values ± standard deviation (SD), and one-way variance analysis (ANOVA) was used to analyze the results obtained, followed by the Tukey test, with a *p*-value of <0.05 considered statistically significant.

### 2.5. Bioactivity Assay Using SBF Solution

In bioactivity analysis, the Ti/Hap-1, Ti/Hap-3, and Ti/HAp-coll were immersed in simulated body fluids (SBF) and kept in an incubator at 37 °C. SBF is the most widely used test medium to investigate material properties such as bioactivity to investigate the formation of an apatite layer on the implant surface to predict bone bioactivity in vivo. The bioactivity assay of the samples was immersed for 3 and 10 days while refreshing the SBF solution every two days [22]. After SBF immersion, the Ti substrate surface was analyzed to identify the deposition of calcium phosphate covering the entire surface, especially in the pores, and to predict bone bioactivity in vivo. In addition, SEM examined the composite surface by energy dispersion spectroscopic analysis (EDS).

The purpose of the composite surface SEM micrograph before SBF immersion is to analyze micro/macropores and microstructure on surfaces of HAp and HAp/Coll nanocomposites integrated into the Ti matrix.

## 3. Results

### 3.1. Characteristics of Hydroxyapatite

**Figure 2:**
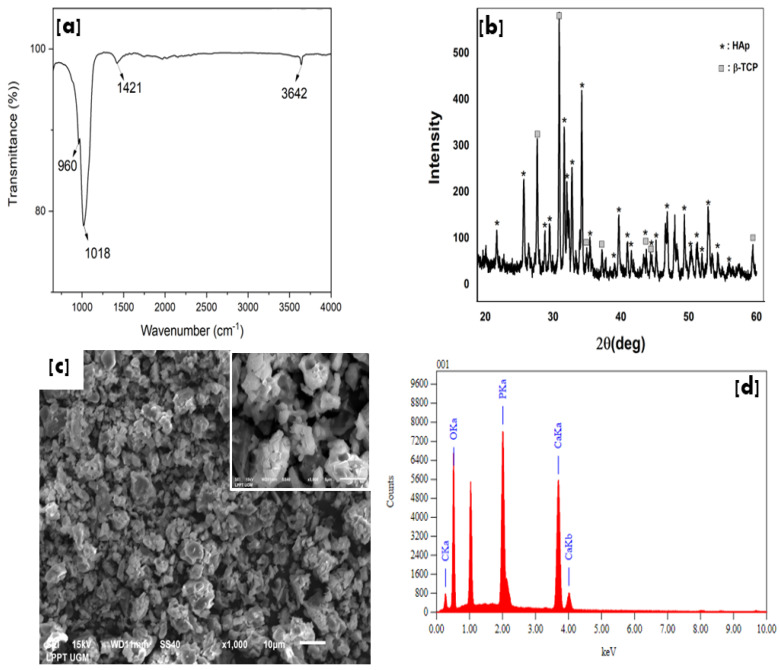
HAp Characteristics of FTIR (**a**), XRD (**b**), SEM morphology with a magnification ×1000 (inset ×5000) (**c**), and EDS spectra (**d**).

Figure 2 shows the characteristic of HAp after calcination at 1000 °C for 2h. The FTIR spectrum (Figure 2a) identified the presence of OH, carbonate, and phosphate groups at 3642 cm^−1^, 1412 cm^−1^, and 1018 cm^−1^ −960 cm^−1,^ respectively [21].

The XRF analysis of the HAp product had a stoichiometry (Ca/P) of 1.72, which is nearly equal to the composition of hydroxyapatite (Ca/P = 1.67). Furthermore, the nanosize HAp has a high crystallinity of 92%. These properties of HAp are very suitable for coating bone implants. The high crystallinity allows the implant to support bone cell growth and does not decompose quickly in body fluids. The HAp fulfills some criteria for coating requirements based on ISO and Food and Drug Administration (FDA) standards, i.e., the stoichiometry range of 1.67–1.76, with a minimum crystallinity of 62% [23].

In addition, XRD spectra of HAp indicated another phase of B-TCP (JCPDS No. 09-0169) [24] with a sharp peak. The SEM morphology of HAp shows agglomerate grains with a porous structure and contains calcium, phosphate, and oxygen based on the EDS spectra.

### 3.2. Characteristics of Ti Substrates Coating

**Figure 3:**
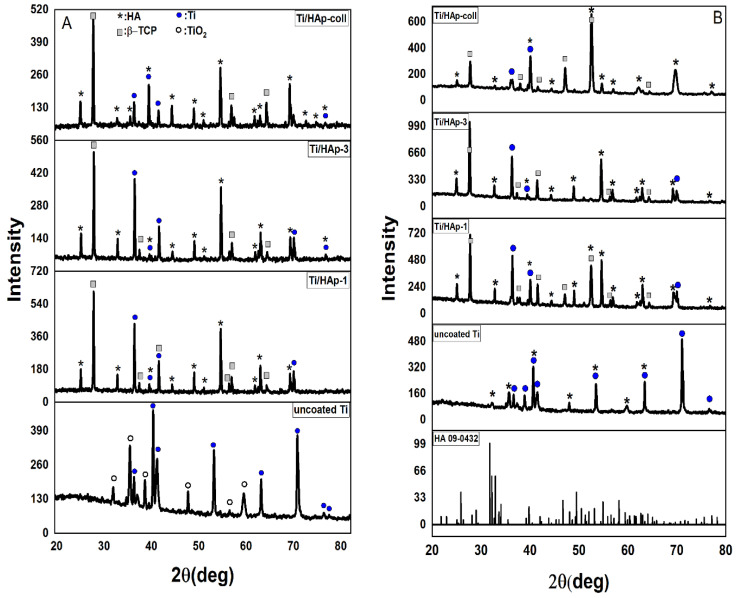
XRD spectra of uncoated-Ti, Ti/HAp-1, Ti/HAp-3, and Ti/HAp-coll coatings before (**A**) and after SBF immersion for 3 days (**B**).

The XRD spectra (Figure 3) of Ti/HAp-1, Ti/HAp-3, and Ti/HAp/Coll show sharp, intense peaks. The diffraction peaks refer to HAp (JCPDS No.09-0432) [25] and Ti (JCPDS no: 89–5009) [26,27]. The crystallite size of Ti/HAp-1, Ti/HAp-3, and Ti/HAp/Coll was 50.31 nm, 42.76 nm, and 36.7 nm estimated from the XRD spectra using the Scherrer equation, with a crystallinity of 63%, 70%, and 66%, respectively.

The XRD spectra of the uncoated-Ti substrate show phases of Ti and titanium dioxide (TiO_2_) (JCPDS no: 84–1286 and 88–1175) [28]. The TiO_2_ phase appeared after the pretreatment with HCl solution at 80 °C for 1 h, then drying at room temperature. The TiO_2_ phase arises due to the thermal treatment of titanium in an oxygen-containing atmosphere. The passive layer formed on titanium consisting of amorphous or poorly crystalline nonstoichiometric TiO_2_ indicates that the Ti substrate is easily reactive in an open oxygen environment [29]. The thermally grown titanium dioxide phases can be anatase, rutile, and brookite, depending on the treatment temperature [30].

**Figure 4:**
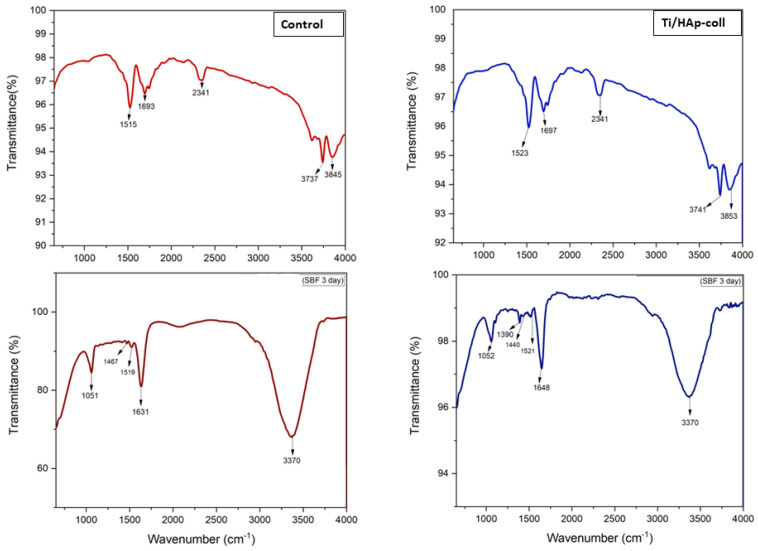
FTIR characteristics of Ti and Ti/HAp-coll substrate before and after SBF immersion (3 days).

Figure 4 shows the FTIR characteristics of uncoated-Ti (after HCl treatment) and Ti/HAp/Coll substrates before and after SBF immersion. The absorption band with moderate intensity of the uncoated-Ti substrate before SBF immersion reveals Ti-OH stretching at 3700–3900 cm^−1^ and OH bending at 1693 cm^−1^ [31,32]. Before SBF immersion, the Ti/HAp-coll absorption bands were similar to the uncoated Ti, indicating that HAp/Coll had been deposited on the Ti substrate after the calcination.

After SBF immersion, the FTIR spectra of the Ti substrate and coating surface show a broad band from 3700 to 2700 cm-^1^, and it is assigned to -OH stretching vibrations from the mixture of metal (Zn, Ti, and Mn) oxides, hydroxides, and phosphates of Ti substrate [33], which has a rough and porous surface after treatment in HCl (SEM morphology).

The FTIR spectra of Ti/HAp-coll substrate before and after immersion in SBF are significantly different. The sintering process causes no traces of collagen in the Ti/HAp-coll due to protein denaturation. However, after SBF immersion (3 days), the traces of amino acids derived from collagen were indicated by absorption peaks at 1631–1648 cm^−1^ and 1519–1521 cm^−1^ corresponding to protein amide I and amide II [34]. The bands at 1440–1467 cm^−1^ correspond to carbonate, and at 1051–1052 cm^−1^ correspond to phosphate [21,35,36]. The presence of organic components in the surface layer is manifested by minor peaks between 3000 and 2800 cm^−1^ and 1500 and 1200 cm^−1^ [37,38].

### 3.3. Compressive Strength

Mechanical strength is an essential factor in developing the scaffold, which ensures that the scaffold must be strong enough to be under mechanical stress from the surrounding tissue. Conversely, the low mechanical strength of the scaffold can affect the changes in the dimensions of the scaffold.

The result of a mechanical test of Ti substrates is summarized in Table 2, and they show that the uncoated Ti (control) has a low compressive strength compared to the Ti/HAp-1, Ti/HAp-3, and Ti/HAp-coll substrates.

**Figure 5:**
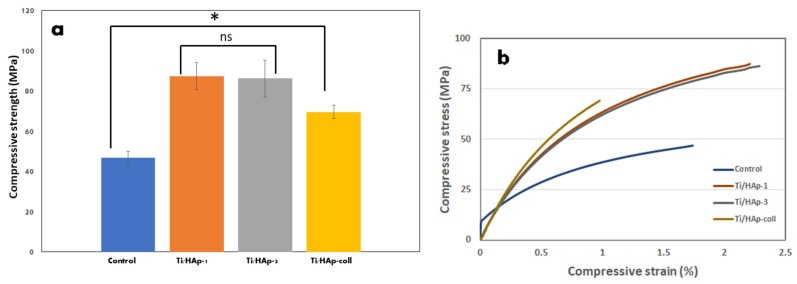
Compressive strength (*: *p* < 0.05) (**a**) and stress-strain curve of coated substrates Ti (**b**).

The mechanical properties of the Ti/HAp and Ti/HAp-coll metal coatings are shown in Figure 5. In Figure 5a, the compressive strength of the Ti/HAp substrate significantly increases compared to the control (*p* < 0.05). The increased compressive strength of Ti/HAp is indicated by the increased surface area/thickness on the Ti/HAp substrate, as confirmed by the SEM morphology. Ti/HAp-1 and Ti/HAp-3 showed that a slight difference in HAp concentration gave a layer thickness and mechanical strength.

The compressive strength of Ti/HAp increased in reference to the uncoated Ti substrate due to the deposition of high crystallinity nanosized HAp on the pores surface of the Ti substrate. The one-way ANOVA analysis showed a significant difference in the compressive strength of Ti/HAp-1, Ti/HAp-3, and Ti/HAp-coll compared to the control.

The stress-strain response of the control, Ti/Hap and Ti/Hap-coll is shown in Figure 5b. The control/uncoated Ti when subjected to a load exceeding its maximum strength, initially exhibits an elastic deformation stage and then exhibits the ductile properties of titanium. The elastic deformation stage in the control was caused by the porous substrate surface and deep spiral holes after HCl treatment. Ti/HAp and Ti/HAp-coll substrates showed ductility, due to the properties of titanium, and the adhesion and agglomeration of HAp and HAp/coll particles (EPD process) and increases the compressive strength of the sample. The strengthening of Ti/HAp and Ti/HAp-coll materials is due to the presence of interfacial bonds between HAp and HAp/Coll nanoparticles on the surface of the Ti substrate.

### 3.4. SEM Morphology of Coating Ti

The EPD of HAp nanoparticles is the subject of current intense experimental efforts [39]. Nanoparticle deposition offers advantages for fabricating ceramic coatings and bodies with solid particle packaging (‘green’ density), good sinterability, and homogeneous microstructure. In addition, the agglomeration of ceramic powders is an essential factor in controlling sintering behavior.

**Figure 6:**
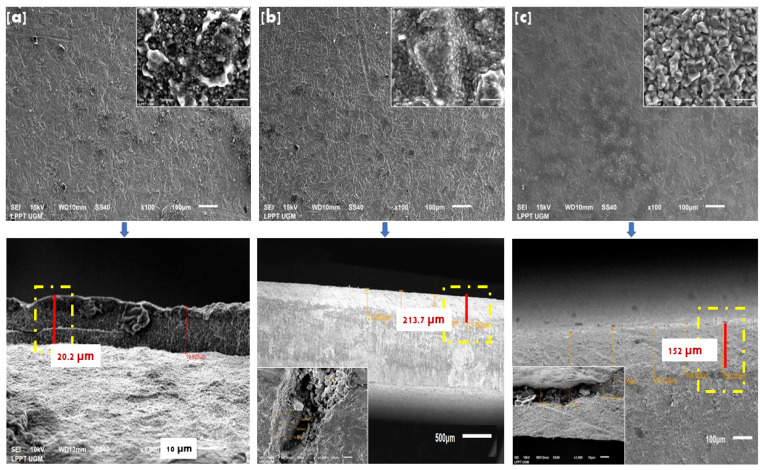
Surface morphology and cross-section (yellow dash-dot line) on substrate Ti/HAp-1 (**a**), Ti/HAP-3 (**b**), and Ti/HAp-coll (**c**).

SEM morphology on Ti/Hap-1 surfaces (Figure 6a), Ti/HAp-3 (Figure 6b), and Ti/HAp-coll (Figure 6c) fabricated using the EPD method (x100 magnification) indicates that HAp coating perfectly covers the surface and does not show cracks after calcination. The inset (magnification of 5000×) shows that Ti/HAp and Ti/HAp/Coll have a crystal size of less than 50 nm. Ti/HAp-coll inset (5000× magnification) shows massive apatite growth due to the addition of collagen, which has a crystal size of more than 1μm. The Ti/HAp substrate surface shows a solid packaging and homogeneous microstructure because the EPD process allows HAp nanoparticles to fabricate ceramic deposits [40].

The SEM micrographs show that the coating thickness of Ti/HAp-1, Ti/HAp-3, and Ti/HAp-coll ranges from 19.62 to 213 µm. Furthermore, the thickness of the Ti substrate coating shows that the EPD method has been used successfully to produce a thick ceramic film of 200 µm [41,42].

### 3.5. Bioactivity of Ti Coating in SBF

The SBF assay is the only chemical model presented as a measure of the biological activity of the implant surface based on the assumption that the nucleation ability of the surface apatite is related to its bioactivity.

**Figure 7:**
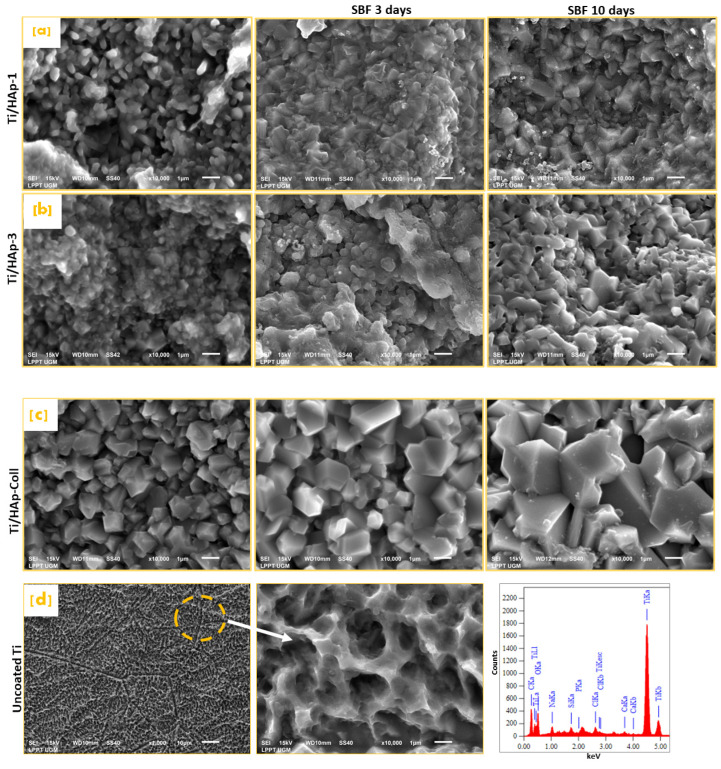
Surface morphology of Ti substrates before and after SBF immersion, Ti/HAp-1 (**a**); Ti/HAp-3 (**b**), Ti/HAp-coll (**c**), and uncoated Ti after 3 days SBF immersion with EDS spectra (**d**).

The SEM morphology of Ti/HAp-1, Ti/HAp-3, and Ti/HAp-coll before SBF immersion is shown in Figure 7. The solid-agglomerate surface on Ti/HAp-3 is caused by the high concentration of HAp (3%), whereas Ti/HAp-1 and Ti/HAp-coll showed a porous surface after the calcination. After 10 days of SBF immersion, as seen in Figure 7, all samples showed a completely enclosed surface morphology with a compact HAp block. The HAp block diameter on Ti/HAp-1 and Ti/HAp-3 was 1–2 μm, and Ti/Hap-coll was 3–4 μm. In addition, the increases in HAp blocks show significant apatite crystal growth through immersed days. Figure 7d shows that the surface of uncoated Ti after three days of SBF is spongy with a pore size of 1 µm.

**Figure 8:**
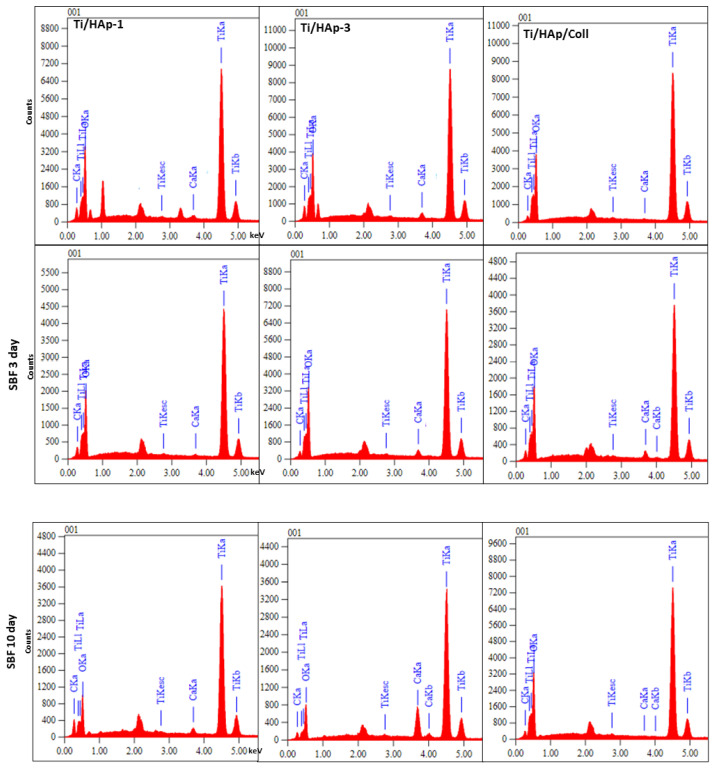
The EDS spectrum of Ti/HAp-1, Ti/HAp-3, and Ti/HAp-coll before SBF and after SBF immersion.

The surface morphology of uncoated-Ti shows a spongy pores size of 2 µm due to pretreatment in HCl solution (Figure 7d). The EDS spectra collected in the selected micro-area on the uncoated Ti surface reveal the peaks of titanium, sodium, phosphate, calcium, and chlorine after being immersed in SBF. The deposition of salts sourced from the SBF solution occurs in the pores on the substrate surface [34,43]. The EDS spectra of the surface area of Ti/HAp and Ti/HAp-coll in Figure 8 show peaks from the substrates, i.e., titanium, calcium, and phosphate.

**Figure 9:**
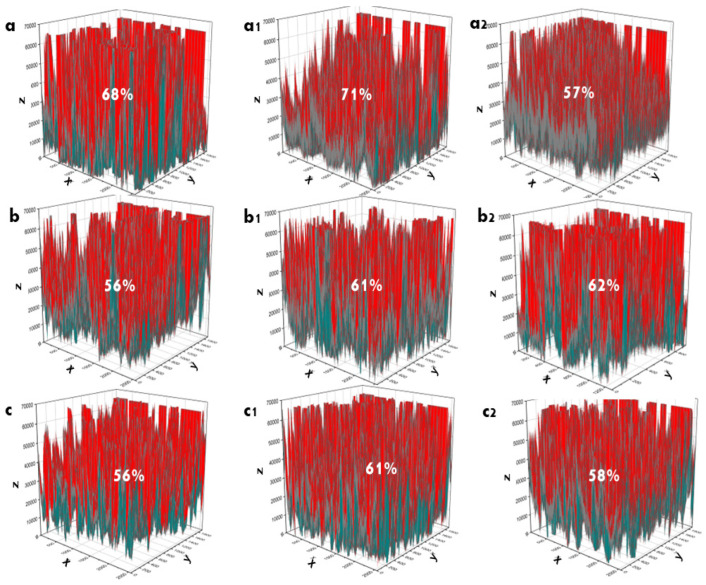
Surface porosity (%) of Ti/HAp-1 (**a**–**a2**), Ti/HAp-3 (**b**–**b2**), and Ti/HAp-coll (**c**–**c2**). Before immersion (**a**,**b**,**c**), 3 days immersion (**a1**,**b1**,**c1**), and 10 days immersion (**a2**,**b2**,**c2**).

The porosity/surface porosity of the coating was determined based on SEM images. The SEM images were first converted into two-dimensional grayscale matrices, and all procedures were performed using the instructions provided by the OriginPro software [10,21,44,45]. The pore distribution and solid volume of coating are evaluated on the base of color distribution; solid volume is shown by red color, and pore distribution is shown by blue color (Figure 9).

## 4. Discussion

The XRD spectra of Ti/HAp and Ti/HAp-coll before SBF immersion showed diffraction peaks of β-TCP, HAp, and Ti. In addition, the intensity of the Ti phase decreased in the Ti/HAp-coll substrate due to the presence of collagen. After the immersion (3 days), XRD spectra of Ti/HAp and Ti/HAp-coll indicate the presence of Ti, HAp, and β-TCP phases. The Ti phase shows decreased intensity due to the growth of apatite at multiple phases of HAp and β-TCP covering the substrate.

FTIR spectrum of Ti/HAp-coll shows the presence of protein amide I and amide II of collagen after the immersion, indicating a protein phosphorylation process that ‘maintains’ collagen even though it has a denaturation protein structure.

The interaction of collagen and HAp in bone has been reported in previous research. Bone has a hierarchical structure based on mineralized fibrils, an organic matrix representing collagen proteins in tight interaction with the mineral hydroxyapatite (HAp) and is stabilized by water molecules. Collagen has functional groups of charged amino acid groups in type I collagen and a high charge density at discrete intrafibrillar sites in macromolecules that can interact with calcium and phosphate ions [46,47].

Accordingly, it is reasonable to assume that collagen interaction with HAp occurs during the EPD process. Therefore, mineralized collagen is due to the exchange of collagen structure with HAp crystals [48,49].

The sintering process of Ti/HAp/Coll denatured the protein in the sample, but the bonding between Coll and HAp protects the protein due to the tight interaction. We are convinced that protein phosphorylation occurred during the HAp/Coll nanocomposite solution preparation in the coating process, where a phosphoryl group was bound to amino acids. Therefore, the phosphate groups and the protein amino acid I present in the FTIR spectra after immersion, due to phosphate, are essential in activating proteins to perform certain cell functions [10,50,51]. Furthermore, Coll type-1 is a template for the binding of calcium and phosphate ions and the subsequent nucleation of apatite crystals [52,53]. This role of coll was proven by the significant apatite growth on the surface of the Ti/HAp/Coll substrate confirmed in SEM data.

After the immersion, the FTIR spectra of the uncoated Ti substrate showed that the SBF solution supports apatite growth on the surface without HAp coating. Natural apatite is one of the biological varieties of calcium phosphate; it is biologically adaptable, occurs in the first 24 h of soaking in SBF solution, and increases to day 7. Apatite produced naturally on the surface of SBF solution can bind bones through a layer of apatite formed on living beings’ bodies [54]. Therefore, as shown in Figure 7d, the surface morphology of uncoated Ti and the EDS spectra indicate the apatite component (Ca and P).

The electric field induced by the EPD allows the bulk of the colloidal particles to migrate to the porous/complex material’s interior, creating a porous surface and micro/nano-controlled topography template. However, the compressive strength of control/uncoated Ti (46.71 MPa) was low compared to previous studies (control/uncoated Ti 83.30 MPa; using the EP2D method) due to different pretreatment on Ti substrates [55,56].

The pretreatment of the Ti substrate with HCl causes a rough and porous surface with spiral depth, which induces low compressive strength in the uncoated Ti. However, rough and porous surfaces on substrates provide excellent adhesion opportunities during the EPD process. The HAp and nanocomposite HAp/Coll particles adhere to and fill rough and porous surfaces up to a thickness of ~200 µm, and agglomeration particles can increase the compressive strength of the substrate. As a result, Ti/HAp and Ti/HAp-coll substrates had a compressive strength range from 69 to 87 MPa and were suitable for trabecular bone (0.2–80 MPa) [57,58].

The EPD process and the Ti substrate sintered at 900 °C increased the thickness and hardness of the substrate coating, as reported by previous studies [23,59]. The thickness of the coating standards ranges from 50 to 200 µm, and the study results show a layer thickness of up to 266 µm. In addition, the concentration of EPD solution also affects the coating thickness [60].

The surface morphology on Ti/HAp-1 and Ti/HAp-3 before SBF showed a micropore size of less than 1 µm. In contrast to macropores, micropores provide greater surface area, favorable adhesion for proteins, and cell attachment to implants in vitro. The pore size <10 µm (micropore) creates a larger surface area and supports greater ion exchange and bone protein adsorption. Bone growth in a scaffold with a micropore structure can fill in the initial empty micropore network and absorb/brittle bone bioceramics. The implant micropore structure improves the implant’s mechanical properties [61].

Porosity and pore size plays an essential role in the degradation process of the scaffold, and greater porosity causes increased permeability, resulting in faster degradation. However, the scaffolds from this study had decreased porosity after immersion in SBF solution due to apatite growth [62,63,64].

Before SBF immersion, Ti/HAp-1 had high porosity (68%), and Ti/HAp-3 and Ti/HAp/Coll had a lower porosity of ~56%. Ti/HAp-3 and Ti/HAp/Coll have low porosity due to the high concentration of HA and collagen deposited on the substrate surface. However, on 3 days of immersion, all samples show an increased porosity of 4–5%, significantly on Ti/HAp-1. After 10 days of immersion, Ti/HAp-1 and Ti/HAp-coll had lower porosity than Ti/HAp-3 due to the significant growth of apatite crystals on Ti/HAp-1 and Ti/HAp-coll, seen on SEM morphology (Figure 7).

The SEM pattern (×10,000) after 3 days of SBF immersion, as shown in Figure 7a–c, indicates that HAp covers Ti surfaces, showed apatite growth compared to the surface morphology before SBF, and had a porous and rough structure. The growth of apatite on the surface proved that HAp is bioactive in forming new crystal apatite.

In addition, apatite growth is more significant on the Ti/HAp-coll substrate due to the effect of collagen, providing more intense apatite growth compared to Ti/HAP-1 and Ti/HAp-3. The EDS spectra before and after SBF immersion shows the elements of oxygen, calcium, phosphate, and titanium. The component intensity of elements changes as a function of the concentration of the coating solution and the time of SBF immersion.

Figure 7d shows that the surface of uncoated Ti has rough and uniform nanopores that help increase the bone contact area for the implant and reduce the healing time from implant surgery [65]. The ×10,000 magnification SEM showed the many uniform nanometer holes with fairly deep spiral structure holes that helped the osteoblast tentacles reach deeper and increase the implant stability after implantation. In addition, pretreatment by HCl provides superior surface modification effects in titanium, reducing roughness and post-etching mass loss [66].

The Ti substrate sintered at 900 °C showed a complex coating surface, uniform layer thickness, and a high deposition rate. In addition, the HAp coating increases the corrosion resistance of the biomaterial as a barrier against metal ions.

## 5. Conclusions

The HAp product synthesized from the *P. maxima* shell has a nanocrystalline size of 32nm, stochiometry (Ca/P) 1.72, and crystallinity (92%); it is excellent for application in coating Ti substrates, according to ISO and Food and Drug Administration (FDA) standards. Coating Ti substrates in the solution of 1 % and 3 % HAp increases the layer thickness. The compressive strength of Ti/HAp-1 and Ti/HAp-3 (86–87 Mpa) increased significantly in reference to the control (uncoated Ti), which is an applicable requirement for trabecular bone material (0.2–80 MPa). Furthermore, the Ti/HAp-coll substrate was 69 MPa, due to the substitution of Coll (1%). The Bioactivity test in the SBF immersion showed significant growth apatite on Ti/HAp-coll substrates. Therefore, Ti/HAp and Ti/ HAp-coll provide excellent compressive strength and intense apatite growth potential as bone implant material.

## Figures and Tables

**Table 1:** Amount of regent for preparing 1000 mL of SBF [16].

No	Reagent	Amount
1	NaCl	8.035 g
2	NaHCO_3_	0.355 g
3	KCl	0.225 g
4	$K_{2}{\mathrm{HPO}}_{4}\cdot 3H_{2}O$	0.231 g
5	${\mathrm{MgCl}}_{2}\cdot 6H_{2}O$	0.311 g
6	1.0 M HCl	39 mL
7	CaCl_2_	0.292 g
8	Na_2_SO_4_	0.072 g
9	1.0 M HCl	0–5 mL
10	Tris (hydroxymethyl) aminomethane	6118 g

**Table 2:** Compressive strength of Ti coating with HAp and bovine collagen nanocomposites.

No	Sample	Force (kN)	Surface Area (mm^2^)	Compressive Strength (MPa)
1	Control (Uncoated Ti)	0.56	11.99	46.71
2	Ti/HAp-1	1.07	12.26	87.28
3	Ti/HAp-3	1.08	12.53	86.19
4	Ti/HAp-coll	0.85	12.26	69.33

## Data Availability

The data presented in this study are available on request from the Corresponding author.
